# Investigating factors of metabolic bone disease in baboons (*Papio* spp.) using museum collections

**DOI:** 10.1002/ajpa.24450

**Published:** 2021-12-02

**Authors:** Srishti Sadhir, Andrea R. Eller, Stephanie L. Canington, Sabrina B. Sholts

**Affiliations:** ^1^ Department of Anthropology, National Museum of Natural History Smithsonian Institution Washington District of Columbia USA; ^2^ Department of Evolutionary Anthropology Duke University Durham North Carolina USA; ^3^ Center for Functional Anatomy and Evolution Johns Hopkins University School of Medicine Baltimore Maryland USA

**Keywords:** captivity, metabolic disease, nonhuman primate, *Papio*, skeletal pathology

## Abstract

**Objectives:**

To assess manifestations of metabolic bone disease (MBD) and their potential environmental and phenotypic factors in captive and non‐captive baboon (*Papio* spp.) specimens.

**Materials and methods:**

Our sample consisted of 160 baboon specimens at the Smithsonian's National Museum of Natural History accessioned from 1890 to 1971. Combining cranial indicators of MBD and the museum's historical data, we examined factors contributing to likely instances of MBD. We used binomial‐family generalized linear models to assess differences in MBD frequency by environment (captive, non‐captive), specimen accession year, and skin color (light, medium, dark).

**Results:**

Indicators of MBD were most frequently observed in captive baboons, with a decrease in MBD frequency over time. Fifteen non‐captive individuals showed indicators of MBD, which are the first published cases of MBD in non‐captive nonhuman primates (NHPs) to our knowledge. The most common MBD indicators were bone porosity (n = 35) and bone thickening/enlargement (n = 35). Fibrous osteodystrophy was observed frequently in our sample, likely relating to nutritional deficiencies. We found no association between exposed facial skin color variation and MBD.

**Conclusions:**

Our findings are consistent with historical accounts of MBD prevalence in captive facilities, especially earlier in the 20th century. A decrease in MBD prevalence later in the 20th century likely reflects improvements in housing, diet, and veterinary care in captive settings. Causes of MBD development in non‐captive baboons should be further explored, as understanding the potential health impacts that anthropogenic environments impose on NHPs is imperative as humans increasingly alter the natural world in the 21st century.

## Graphical abstract

Baboon crania that exhibit variation in metabolic bone disease presentation (b, c), compared to a cranium without disease (a).

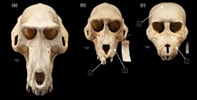

## 1 INTRODUCTION

Metabolic bone disease (MBD) encompasses a range of pathological conditions of bone formation, remodeling, and/or mineralization that can include rickets, osteomalacia, and fibrous osteodystrophy (Canington & Hunt, 2016; Olson et al., 2015; Uhl, 2018; Wharton & Bishop, 2003). This group of conditions is characterized by changes in bioavailable mineral quantities (e.g., calcium or phosphorus), deficiencies in nutrients such as vitamin D, and/or organ dysfunction (Craig et al., 2016; Uhl, 2018; Wolfensohn, 2003). MBD is found across terrestrial vertebrates (Craig et al., 2016), including humans and nonhuman primates (NHPs) (Farrell et al., 2015; Hatt & Sainsbury, 1998; Rajakumar, 2003; Wharton & Bishop, 2003; Wolfensohn, 2003), other mammals (Chesney & Hedberg, 2010; Uhl, 2018), and amphibians and reptiles (Klaphake, 2010). Diet, sunlight exposure, stressful environments, renal disease, genetic factors, and hormonal abnormalities have all been implicated in multifactorial MBD etiology (Farrell et al., 2015; Hannan et al., 2019; Uhl, 2018). Though speculative, gastrointestinal parasites may also play a role in MBD etiology, as noted in in camels (Lynch et al., 1999) and reptiles (Loukopoulos et al., 2007). Osteological markers of MBD include gross bone structural changes, reduced mineral density, and poorly formed cortices and trabeculae, all of which can be regionally localized or found across the skeleton (Adkesson & Langan, 2007; Farrell et al., 2015).

The general diagnosis of “metabolic bone disease” is commonly used instead of specific diagnoses (such as rickets, osteomalacia, or fibrous osteodystrophy) or when multiple conditions are present in an individual (Uhl, 2018). Of all MBDs, fibrous osteodystrophy appears most prominently in the cranium and mandible, whereas rickets and osteomalacia are better detected through postcranial elements (Table 1). The bilateral enlargement of fibrous osteodystrophy lesions in the cranium and mandible is a direct result of osteoclastic bone resorption; bone mineral (calcium and phosphorus) is transferred back to the circulatory system, and distortion of the remaining cancellous bone occurs (Canington & Hunt, 2016; Olson et al., 2015). Rickets represents a softening of the bones linked to vitamin D deficiency during development, which occurs prior to epiphyseal fusion (called osteomalacia during adulthood after epiphyseal fusion has ceased; Wharton & Bishop, 2003).

**TABLE 1:** Definitions of specific MBD conditions that may be evident in the study sample, following Canington and Hunt (2016), Craig et al. (2016), Fiennes (1974), Farrell et al. (2015), and Uhl (2018)

MBD condition	Definition
Rickets	Developmental bone disease related to vitamin D and/or phosphorus deficiencies and characterized by poor endochondral ossification at epiphyseal growth plates and poor bone formation, often warping bone structure where there is osteoid but no bone mineral formation; similar to osteomalacia but only affects young animals; if persistent in growth, permanent skeletal changes carry on into adulthood
Osteomalacia	Bone disease related to vitamin D and/or phosphorus deficiencies and changes in modeling and remodeling, where poor bone formation causes lesions similar to rickets and osteoid forms but not bone mineral; does not affect any cartilaginous growth plates because this disease is found in adults only
Fibrous Osteodystrophy	Lesions formed by bone resorption and replacement with immature bone that lacks proper structure and mineralization, results from primary or secondary hyperparathyroidism; secondary hyperparathyroidism is more common and is due to renal disease, elevated parathyroid hormone, and/or calcium and phosphorus mineral imbalance; bilateral enlargement of the cranium and mandible are especially noted with this condition

While genetic factors may play a role in MBD etiology, nongenetic factors are most often implicated (Farrell et al., 2015; Hannan et al., 2019; Uhl, 2018). MBD presence in animals can be particularly revealing about their environmental conditions during life, including anthropogenic (human constructed or influenced) spaces. Many animals are not equipped to handle the novel, rapidly imposed pressures of some anthropogenic environments, which can alter diet, mobility, social behavior, and reproduction (e.g., primates and carnivores; Michalski & Peres, 2005). MBDs are rarely recorded in non‐captive (i.e., “wild”) populations, although nutritionally related MBD has been reported in wild birds, possibly due to a calcium‐deficient diet related to range expansion into suboptimal anthropogenic habitats (Cousquer et al., 2007; Phalen et al., 2005). MBD is well recognized in many domesticated animals (including sheep, goats, llamas, alpacas, cattle, pigs, horses, reptiles, cats, and dogs; Dittmer & Thompson, 2011), frequently linked to deficiencies in nutrition and/or sunlight exposure (Dittmer & Thompson, 2011; Uhl, 2018). For animals in captive settings, including zoological parks and research facilities, MBD has long been associated with nutritional and environmental stress (Fiennes, 1974; Mann, 1930; Ratcliffe, 1966; Wackernagel, 1966).

Historical reports of MBD are confounded by a lack of pathophysiological knowledge and consensus on diagnosing zoo animal pathologies. Instead, all MBDs were broadly classified as “rickets.” At the London Zoo in 1890, surgeon John Bland‐Sutton noted that “half the monkeys and lemurs brought to this country die rickety” (Bland‐Sutton, 1895:266). However, the description of “rickets” in the historical sense more broadly encompassed a suite of MBDs, rather than the specific diagnosis used today. Some historical cases, like “rickets” in lion cubs at the London Zoo in 1889, were most likely fibrous osteodystrophy, where lesions form by bone resorption and replacement with immature bone that lacks proper structure and mineralization (Table 1; Chesney & Hedberg, 2010).

While misdiagnoses were common, historical reports also highlight efforts to change zoological practices for disease prevention; in particular, changes to diets, enclosures, and other animal welfare strategies helped practitioners mitigate MBD prevalence. The first successful treatment of MBD occurred in the aforementioned lion cubs at the London Zoo, after doctors prescribed a diet of goat meat, crushed bones, and cod liver oil. This diet is now known to have a high nutrient content of calcium, phosphorus, vitamin A, and vitamin D, which reversed MBD in these animals (Chesney & Hedberg, 2010). By the 1930s, diet and sunlight had been identified as the key to disease prevention in captive settings (Fiennes, 1974; Mann, 1930; Ratcliffe, 1966; Wackernagel, 1966). As enclosures shifted from restrictive cages to naturalistic designs, zoo veterinarians began to remedy the high prevalence and severity of MBD through dietary means such as vitamin D, calcium, and phosphorus supplements, as well as UV light therapy to produce cutaneous vitamin D (Fiennes, 1974; Ratcliffe, 1966; Wackernagel, 1966). Since animal welfare legislation was enacted in the mid‐20th century (Hosey et al., 2009), and alongside greatly improved diets, habitats, and veterinary care practices (Fiennes, 1974; Gutierrez et al., 2021; Smithsonian Institution, 1942, 1952, 1957), MBDs have been greatly reduced in captive NHPs, save a few isolated cases (Hatt & Sainsbury, 1998; Morrisey et al., 1995; Wolfensohn, 2003).

Modern studies of MBDs in NHPs have led to a better understanding of specific MBD pathways and attributes and highlighted the importance of improved captive conditions. Farrell et al. (2015) compared a large sample of captive NHP crania from historical (late‐19th to mid‐20th century) and recent (1980s and onwards) collections and observed the absence of osteomalacia and other forms of MBD with the modernization of captive NHP care. Baboons showed one of the highest frequencies of MBD among the 12 taxa studied, for example, 8.2% in *Papio* versus 3.5% in the total sample (Farrell et al., 2015), although the “natural baseline” of MBD in non‐captive populations is unknown. Notably, baboons display strong interspecies variability in skin and pelage morphologies, including pigmentation (Hill, 1967; Kamilar, 2006). In both non‐captive and captive living baboons, exposed skin color influences vitamin D_3_ production, but not downstream metabolism (Ziegler et al., 2018). Because of the known connection between sunlight and MBD, it is plausible that skin color differences could lead to variable disease outcomes, as documented in humans (Holick, 2004). In addition, the relative availability of dietary vitamins and minerals, and/or the functionality of their physiological pathways, may also contribute to variable disease outcomes. As far as is known, all catarrhine primates, including *Papio* baboons, have similar requirements for vitamins and minerals (Milton, 2003), including calcium and phosphorus, and similar levels of circulating parathyroid hormone (Fincham et al., 1993), all of which can cause MBD if imbalances or disruption to physiological pathways occur. There are some physiological differences within the Order Primates specifically related to vitamin D, for example, differential synthesis rates between vitamin D_2_ and vitamin D_3_ and different levels of circulating vitamin D (Crissey et al., 1999; Gacad et al., 1992; Marx et al., 1989). These differences are most pronounced between platyrrhine and the vitamin D_3_‐resistant catarrhine primates, the latter having very low vitamin D uptake by target cells (i.e., cell “resistance”) resulting in higher levels of circulating bioactive vitamin D (Adams et al., 1985; Gacad et al., 1992).

In this study, we investigated cranial and mandibular indicators of MBD in baboons (*Papio* spp.) to better understand the potential health impacts of anthropogenic environments on NHPs. Past studies of MBD have predominantly focused on pathological analysis and approaches to remedying animal disease (e.g., Fiennes, 1974; Ratcliffe, 1966; Wackernagel, 1966). However, we take an ecological approach to the study of MBD, considering how diseases can arise when animals are subjected to environmental conditions that differ from their natural habitat (Eller et al., 2019). Using a large sample of non‐captive and captive baboon cranial and mandibular specimens spanning nearly a century (1890–1971) at the Smithsonian Institution's National Museum of Natural History (NMNH), we expand on work by Farrell et al. (2015) and Ziegler et al. (2018) by examining environmental and physiological factors and skeletal manifestations of MBD. We hypothesize that anthropogenic environments are conducive to MBD in NHPs. Accordingly, we test three predictions: 1) the captive group has a higher MBD frequency than the non‐captive group, 2) the difference in MBD frequency between captive and non‐captive groups decreases as captive conditions improve over time, and 3) skin color groups do not differ in MBD frequency, where baboons with darker facial skin color do not have a higher incidence of MBD than baboons with lighter facial skin color. This final prediction addresses the complexity in MBD etiology. Both skin color (as a proxy for cutaneous vitamin D synthesis; Ziegler et al., 2018) and diet (vitamins and minerals) influence MBD etiology, although the latter is not tested here.

## 2 MATERIALS AND METHODS

### 2.1 Sample

We macroscopically examined 160 baboon (*Papio* spp.) skulls in the Division of Mammals at the NMNH (Supplementary Table 1). Due to the over‐representation of primate skulls in the NMNH collections relative to postcranial elements, we focused on cranial and mandibular manifestations of MBD to allow for a larger sample size. Table 2 summarizes the demographic information collected from specimen tags and NMNH accession records for each specimen. Taxonomic designations followed the Zinner et al. (2011) scheme, which include six distinct *Papio* species: *P. anubis*, *P. papio*, *P. ursinus*, *P. cynocephalus*, *P. kindae*, and *P. hamadryas*.

**TABLE 2:** Taxonomic distribution of specimens by skin color group, sex, age, accession year, and environment type

Taxon	Total	Color	Sex		Age				Accession year		Environment	
			Female	Male	Infant	Juvenile	Subadult	Adult	1890–1949	1950–1971	Captive	Wild
*P. h. anubis*	69	D	31	38	8	6	9	46	29	40	45	24
*P. h. cynocephalus*	24	M	14	10	1	3	5	15	2	22	19	5
*P. h. hamadryas*	9	L	3	6	1	3	2	3	7	2	9	0
*P. h. papio*	17	D	2	15	0	6	1	10	2	15	11	6
*P. h. ursinus*	41	M	17	24	2	4	7	28	24	17	4	37
Total	160	n/a	67	93	12	22	24	102	64	96	88	72

*Note*: Specimens were classified as “captive” if the individual lived in a captivity at any point during life, regardless of birthplace. Accession years were grouped for descriptive purposes but not for statistical testing.

Environmental assignments were based on whether an individual died in their native African habitat (“non‐captive”) or in captivity (“captive”), including zoological parks and biomedical facilities. Non‐captive individuals were further geographically and temporally subdivided to consider habitat diversity across Africa (Supplementary Table 2). Additionally, the specimen's date of accession (the most reliable date available) was recorded from each tag, indicating the year that NMNH acquired the remains, usually soon after death (Supplementary Table 3).

Age was estimated using molar eruption stages following Kahumbu and Eley (1991) and Phillips‐Conroy and Jolly (1988). Each specimen was assigned to one of four categories: infant (no molars fully erupted, <20 months old), juvenile (all first molars completely erupted, 20–49 months old), subadult (all second molars completely erupted, 50–80 months old), or adult (all third molars completely erupted, >80 months old). Molar eruption is more strongly correlated with age than are other tooth eruption sequences or cranial characteristics (e.g., sutural fusion and cranial size), all of which may confound aging estimates because of their relationship with sex and social dominance rank (Galbany et al., 2015; Phillips‐Conroy & Jolly, 1988). For the purposes of this study, subadult describes an individual approaching reproductive maturation. Sex was primarily determined from the specimen tag but also assessed by canine size, a highly sexually dimorphic feature in adult baboons (Phillips‐Conroy & Jolly, 1981).

**FIGURE 1:**
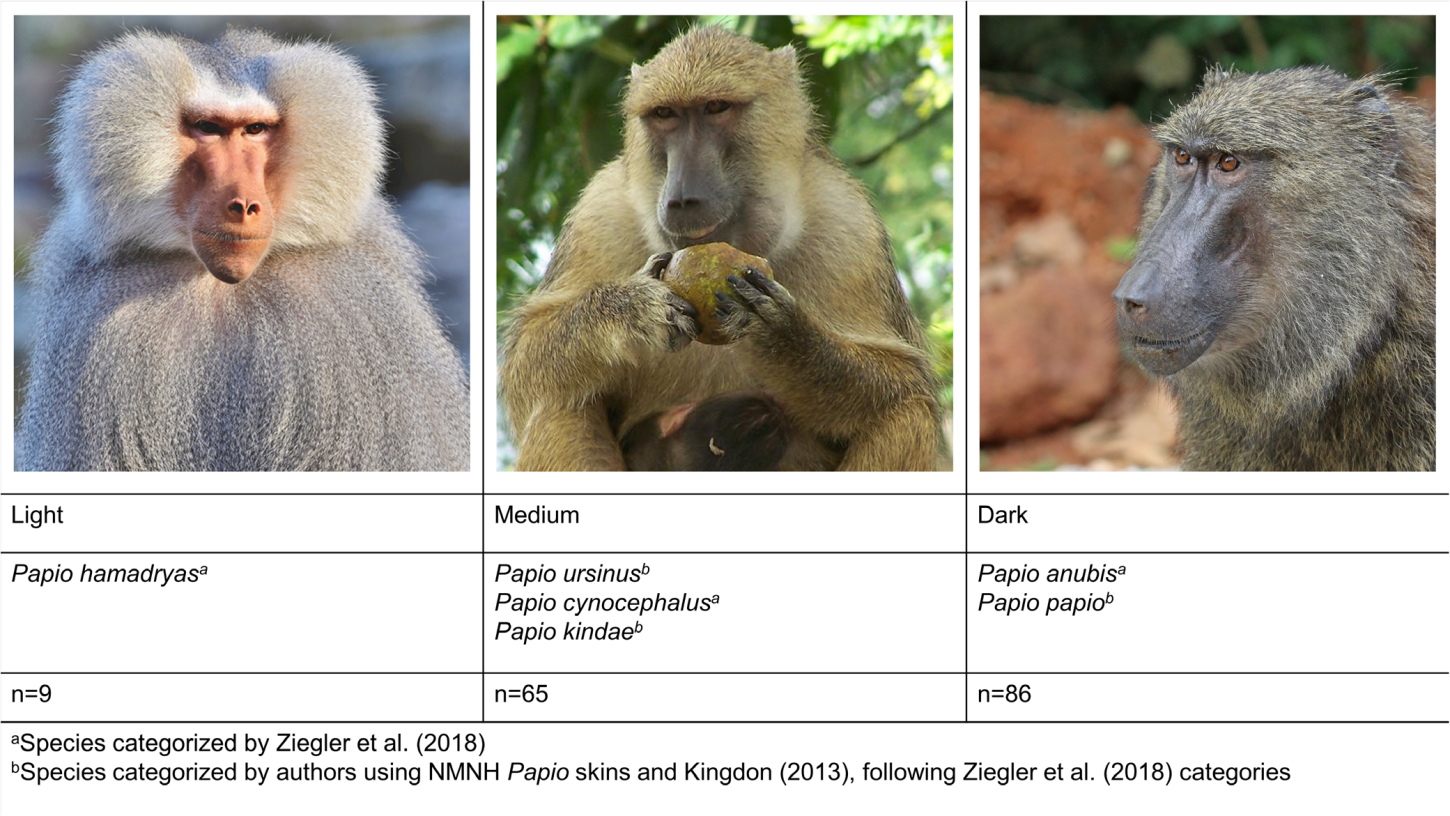
Skin color categories used to assess the influence of skin color on cranial and mandibular indicators of MBD. (Image credits: Pixabay [left]; Alexander Landfair, University of Dar es Salaam via Wikipedia [middle]; Sharp Photography via Wikimedia Commons [right])

Facial skin pigmentation was classified into three color groups: light, medium, and dark (Figure 1). Following Ziegler et al. (2018), three baboon species were categorized in their visually determined color scale: *P. anubis* as dark, *P. cynocephalus* and *P. kindae* as medium, and *P. hamadryas* as light. For *P. papio* and *P. ursinus*, we used Kingdon et al. (2013) for skin and pelage pigmentation descriptions to place *P. papio* in the “dark” category and *P. ursinus* in the “medium” category. When possible, associated specimen skins were visually assessed to confirm the group assignment.

### 2.2 MBD assessment

**FIGURE 2:**
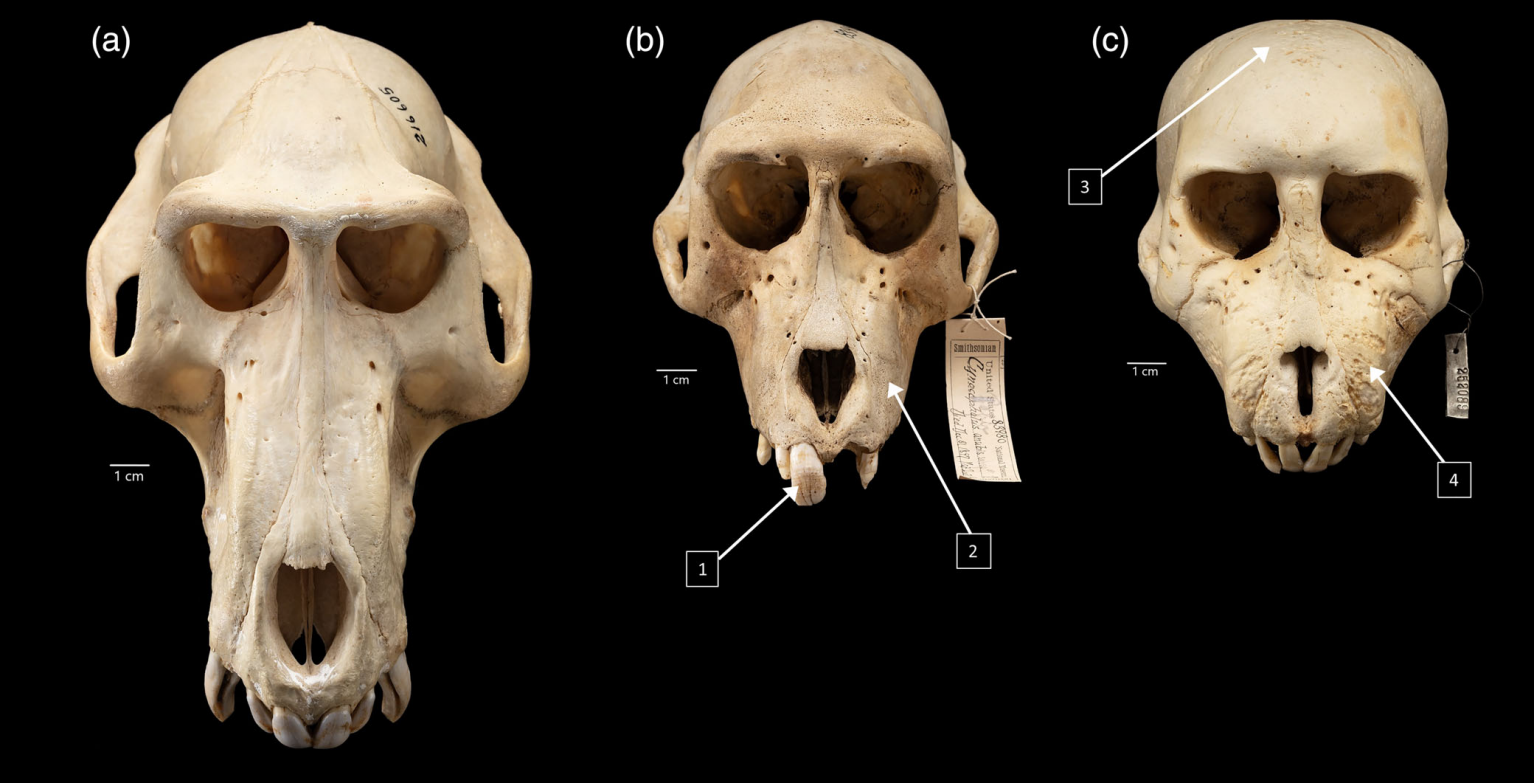
Baboon specimens in the study sample that exhibit variation with respect to MBD criteria, from complete absence to multiple indicators consistent with fibrous osteodystrophy among other possible MBDs. (a) non‐captive *P. anubis* individual (USNM 216605) with no cranial evidence of MBD. (b) Captive *P. anubis* individual (USNM 83980) with minor MBD pathologies including (1) enamel hypoplasia of the incisors and canines, (2) very slight enlargement of the maxilla, and (2) porosity along the maxilla, supraorbital tori, and glabella. (c) Captive *P. ursinus* individual (USNM 252089) with extreme MBD pathologies including (3) extreme porosity, pitting, and enlargement of the neurocranium and (4) maxilla with medially oriented incisors and canines. (Photo credit: Lucia RM Martino, Smithsonian Institution)

Published photos and gross descriptions of MBD cranial indicators in humans and nonhuman primates and domesticated animals provided the basis for our skeletal assessments (Canington & Hunt, 2016; Craig et al., 2016; Farrell et al., 2015; Fiennes, 1974; Uhl, 2018). Because of the longstanding confusion on specific MBD conditions, modern definitions relevant to the current study are provided in Table 1, following Craig et al. (2016). Although MBD does present postcranially, there are far fewer postcranial skeletons available for study at the NMNH (n = 9), and thus only cranial and mandibular evidence was considered. As MBD broadly encompasses a wide range of conditions, we identified a series of pathological criteria, any of which can be indicative of MBD by visual assessment (Figure 2; Table 3). MBD pathological designations were made by agreement of all authors.

**TABLE 3:** Pathological criteria used to identify MBD in the study sample

MDB criteria	References	Observed in sample (n)
Bone porosity in any of the following skull regions: sagittal suture, glabella, and maxilla often associated with pitting; mandible associated with swollen and spongy look; general increase in bone porosity	Farrell et al., 2015	35
Bone thickening and enlargement in any of the following skull regions: frontal, parietal, and/or temporal bones of the cranium; zygomatic bones extending anteriorly into maxilla; maxilla with bilateral enlargement from alveolar margins to frontal process; mandible; nasal conchae; and palate	Canington & Hunt, 2016; Farrell et al., 2015; Fiennes, 1974; Uhl, 2018	35
Mandibular condyles are underdeveloped and/or coronoid processes are missing	Canington & Hunt, 2016; Farrell et al., 2015	10
Rounded orbital margins with softened orbital edge	Farrell et al., 2015	8
Eruption and position of teeth affected when maxilla and mandible has swelled	Farrell et al., 2015; Fiennes, 1974	4
General decrease in bone density	Canington & Hunt, 2016; Farrell et al., 2015	3
Linear enamel hypoplasia in teeth	Guatelli‐Steinberg and Skinner, 2000	4
Fragile, friable, and/or brittle bone regions with inner trabeculae exposure in some cases	Canington & Hunt, 2016; Farrell et al., 2015	2
Lesions of the skull	Uhl, 2018	1
Widening and distortion of cranial and/or facial sutures, almost disarticulated in some cases	Farrell et al., 2015	0
Bowing of mandibular rami	Canington & Hunt, 2016	0

**TABLE 4:** Distribution of MBD pathologies between the skin color categories, including light, medium, and dark categories

Color	MBD	No MBD	Total	% MBD
Light	7	2	9	77.8
Medium	20	45	65	30.8
Dark	24	62	86	27.9
Total	51	109	160	31.9

### 2.3 Statistical analysis

We used binomial‐family generalized linear model (GLMs) to assess differences in MBD frequency by environment (captive, non‐captive), specimen accession year, and skin color. The binomial‐family GLM uses logistic regression analysis to predict a binary outcome based on explanatory variable(s); the binary outcome in this analysis is presence or absence of MBD. For the specimen accession year analysis, we built two additional binomial‐family GLMs, one for the captive specimen subsample (n = 88) and one for the non‐captive specimen subsample (n = 72), to assess temporal changes in MBD frequency within each environmental condition. Age, sex, and species were also included as covariates in the five GLMs, examining MBD frequency by: environment, specimen accession year, and skin color. Finally, environment was included as a covariate in the accession year and skin color GLMs. All analyses were conducted with the R statistical programming language (R Core Team, 2021).

## 3 RESULTS

Most of the captive baboons in our sample lived and died in the Smithsonian's National Zoo and Conservation Biology Institute (NZP) in Washington, DC (n = 26; 29.5%) and the Southwest Foundation for Research and Education in San Antonio, Texas (n = 61; 69.3%). One specimen came from the Oregon Regional Primate Center (Beaverton, OR) and one from the Barnum and Bailey Circus. The non‐captive baboons in our sample are predominantly from southern and eastern African localities (n = 62; 86.1% of non‐captive baboons) while the remainder are from central and western Africa (n = 10; Supplementary Table 2). Skeletal indicators of MBD for each specimen are reported in Supplementary Table 4. Of the 160 specimens, 51 (31.9%) showed evidence for MBD pathologies, with the highest pathological prevalence related to bone thickening, enlargement, and porosity of different regions of the skull (Figure 2; Table 3).

**TABLE 5:** Age distribution of specimens by environment

	Captive		Non‐captive	
	Total	% MBD	Total	% MBD
Infant	5	60.0	7	28.6
Juvenile	16	81.3	6	16.7
Subadult	9	66.7	15	13.3
Adult	58	25.9	44	20.5
All	88	42.0	72	19.4

*Note*: Frequency of MBD is noted for each age group.

**FIGURE 3:**
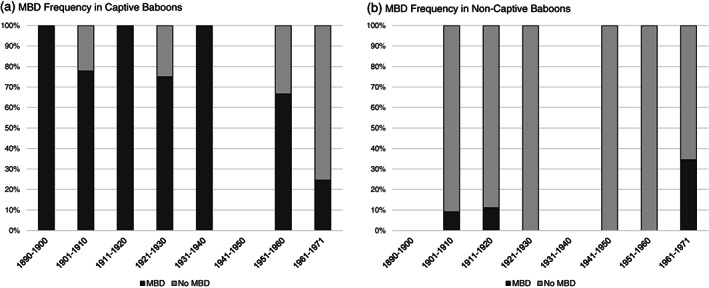
Temporal change in MBD frequency by decade from 1890–1971. Frequencies are expressed as percentages, and there was a high variance in sample sizes across decade bins. (a) MBD frequency in captive baboons decreases over time. (b) MBD frequency in non‐captive baboons does not change over time. Blank decades do not have any represented specimens

Of the 51 pathological individuals, 14 died in their native habitats (killed during hunting and specimen collection trips), while 37 died in captivity. For three individuals, the environment is unknown. Within the total sample (N = 160), captive and non‐captive specimens differed significantly in MBD frequency (LRT: χ^2^ = 8.0, p < 0.01) with captive specimens showing a higher frequency of MBD (42%; Figure 3a) than non‐captive specimens (19%; Figure 3b). MBD frequency decreased significantly among captive baboons over time (1890–1971; LRT: χ^2^ = 6.5, p = 0.01; Figure 3a). In non‐captive baboons, there was no significant change in MBD frequency over time (1890–1971; LRT: χ^2^ = 2.2, p = 0.14; Figure 3b). The 51 specimens with MBD pathologies represent five of the six species, excluding *P. kindae*, and all three categories of exposed facial skin pigmentation (Figure 1). Facial skin color did not significantly predict MBD frequency (LRT: χ^2^ = 5.6, p = 0.06). MBD was not successively more frequent among the “medium” and “dark” groups as compared to the “light” group (Table 4). In the three statistical models examining MBD frequency by environment, specimen accession year, and skin color, age was a significant predictor of MBD (χ^2^ = 10.3–12.9, p < 0.05; Table 5), while sex (Table 6) and species designation were not.

**TABLE 6:** Sex distribution of specimens by environment

	Captive		Non‐captive	
	Total	% MBD	Total	% MBD
Female	37	29.7	30	16.7
Male	51	51.0	42	21.4
All	88	42.0	72	19.4

*Note*: Frequency of MBD is noted for each sex.

## 4 DISCUSSION

### 4.1 MBD indicators and disease specifications

The most frequently identified pathology within our sample was the enlargement and thickening of cranial and mandibular regions. Many of these lesions are consistent with skeletal manifestations of fibrous osteodystrophy, formed by calcium and phosphorus imbalance (Figure 2; Table 1; Bandarra et al., 2011; Canington & Hunt, 2016; Craig et al., 2016; Long et al., 1975; Lynch et al., 1999; Olson et al., 2015). Four of the pathological individuals in our sample, accessioned between 1890 and 1905, have associated accession records indicating rickets. It is likely that these historical diagnoses are not strictly rickets and could represent other MBDs, as the pathophysiology of these conditions was relatively unknown to early researchers. The individuals that are noted to have had rickets (i.e., some form of MBD) were classified in this study as infants, juveniles, and subadults, indicating that individuals with MBD pathologies died before reaching adulthood. These results also point to the rapid and severe nature in how MBD develops even in young individuals, which sets this group of conditions apart from chronic, degenerative diseases often only associated with old age. Osteomalacia was not noted in any historical records linked to the specimens studied.

### 4.2 Captive and non‐captive groups differ in MBD frequency and have more pronounced differences earlier in the 20th century

Our results strongly suggest that the anthropogenic environments of captivity can play an important role in the development of MBD. Overall, rates of MBD were very common in captive individuals and infrequent in non‐captive individuals. Our hypothesis that captive environments are conducive to MBDs in NHPs is thus supported.

In our study sample, 61 specimens came from the Southwest Foundation and 26 from the NZP, with individuals either born at these sites or introduced during life. The first record of baboons at the NZP, specifically *P. cynocephalus*, is noted in the 1893–1894 Annual Report of the Board of Regents of the Smithsonian Institution (Smithsonian Institution, 1894). Over the next 70 years, species were introduced from both non‐captive African settings and other captive settings, including circuses and private homes. In the 1940s, the NZP began noting baboon births, confirming that multiple generations had lived at the zoo since its inception. The last record of the NZP housing baboons was in 1963 when chacma (*P. ursinus*) and olive (*P. anubis*) baboons were gifted to the zoo (Smithsonian Institution, 1964).

In 1960, following the discovery of baboons as model organisms for the study of atherosclerosis, the Southwest Foundation opened and began housing captive NHPs for biomedical research (VandeBerg et al., 2009). Through the 1960s, baboons were trapped in southeastern Kenya and shipped to the Southwest Foundation for its growing colony (Strong & McGill Jr., 1967; VandeBerg et al., 2009). Over the next 60 years, approximately 19,000 baboons were managed in this facility, with the colony continuing into its sixth generation today (VandeBerg et al., 2009).

**FIGURE 4:**
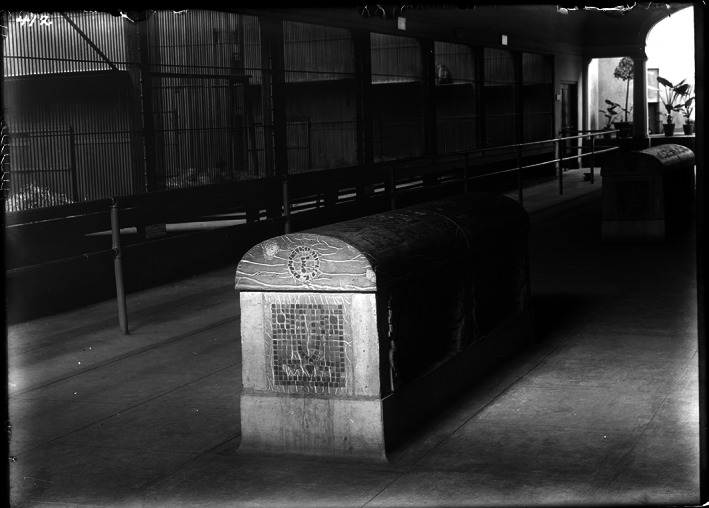
Interior view of the monkey house at the National Zoological Park in 1910 (Smithsonian Institution Archives, 1910). Enclosures consist of small cages with little natural sunlight, no greenery, no temperature control, and no animal enrichment. In the following years, all primates were moved to a new monkey house that was cleaner and had better ventilation, but it was not until the 1950s that better funding allowed for greatly improved conditions. (Image credit: Smithsonian Institution Archives, NZP‐0412)

While both sites are considered captive environments, they differ in many aspects stemming from institutional goals. The NZP, like most zoological parks, was developed with several objectives in mind, including conservation, scientific research, public education, and recreation, while the Southwest Foundation was founded with the sole purpose of biomedical research. As such, the Southwest Foundation attempted to replicate a natural setting in their baboon colony when individuals were not undergoing biomedical trials (as known from the inception of other primate research centers at the time; Yaeger, 1968). Meanwhile, the NZP created small enclosures and modified social groups, sometimes even grouping multiple species from different habitats together, to cater to the public for the purposes of public recreation and science communication (Figure 4). Possibly for this reason, nearly all specimens derived from the NZP showed evidence of MBD, while very few from the Southwest Foundation did. A rich record of NZP documents from the NMNH collections databases and Smithsonian Institution Archives supports the cranial evidence. These records reveal malnutrition, inadequate veterinary care, and poor housing conditions though the mid‐20th century, all which may have contributed to the high prevalence in MBD (Gutierrez et al., 2021).

**FIGURE 5:**
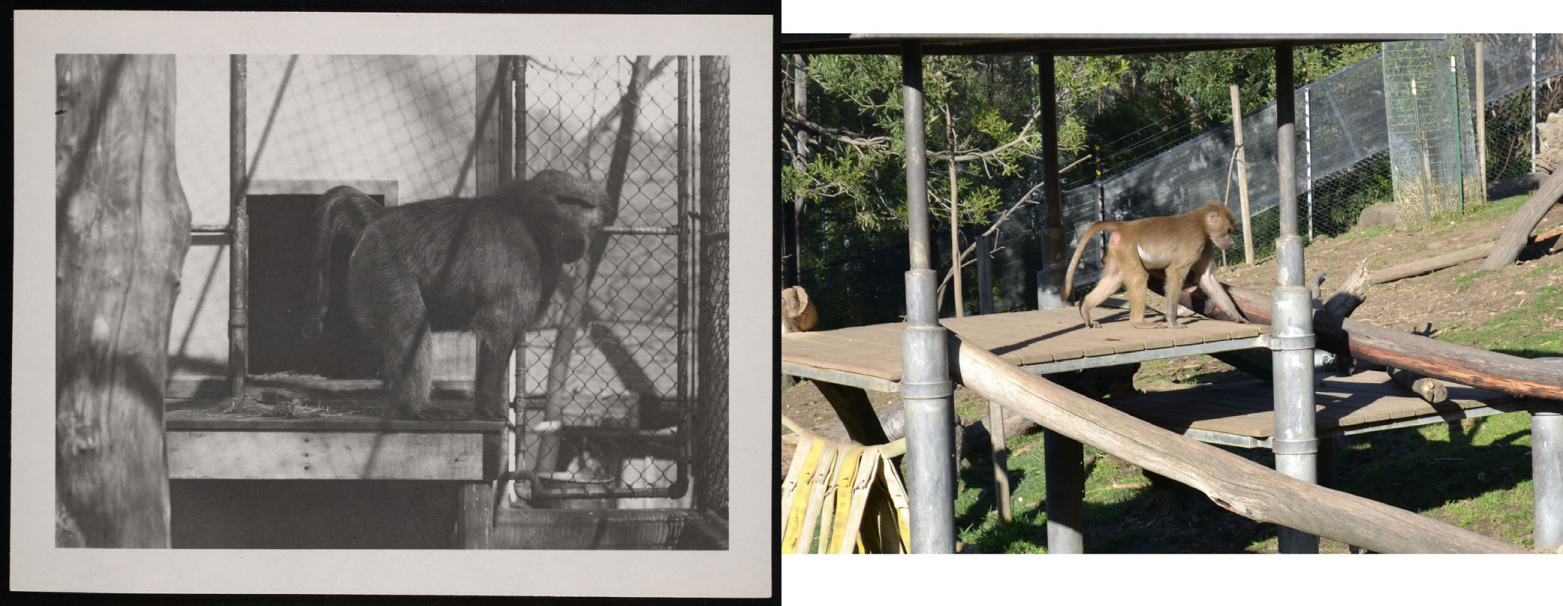
Baboons in historical and modern zoo‐captive environments. (Left) Chacma baboon (*P. ursinus*) at the National Zoological Park in 1935 (Smithsonian Institution Archives, 1935). The enclosure has visibly less naturalistic conditions, including limited space, artificial shade, and little greenery. (Image:). (Right) Hamadryas baboon (*P. hamadryas*) at the Oakland Zoo (Oakland, CA) in 2016. The enclosure is fully outdoors with abundant space, full sunlight exposure, and greenery. (Image credits: Smithsonian Institution Archives, SIA_000095_B48_F13_013 [left]; Oleg Alexandrov via Wikimedia Commons [right])

A complementary explanation is that captive animal care practices had improved by the time the Southwest Foundation opened in 1960. MBD was very common in captive NZP individuals that were accessioned into the NMNH collection earlier in the 20th century, but the rate of MBD prevalence decreased in the subsequent decades with the inclusion of additional Southwest Foundation specimens. It is possible that with better animal welfare practices, captive animals experienced healthier environments later in the 20th century (Figure 5). Our results showed that differences between captive and non‐captive baboon populations became nonexistent into the 1960s and 1970s, a pattern driven by decreasing disease prevalence in the captive group over time. Within the non‐captive group, there was no evidence for a temporal change in MBD frequency.

To our knowledge, this study is the first to identify MBD in non‐captive NHPs. We identified a total of 14 non‐captive baboon specimens with evidence of MBD, spanning 1905 to 1967 (Supplementary Table 1). This result is interesting, but at this time we cannot do more than speculate as to the cause. Under wild conditions, baboons are susceptible to MBD likely through an under‐nourishing diet or possibly through acquired gastrointestinal parasites that alter nutrient uptake (as seen in non‐primate vertebrates; Loukopoulos et al., 2007; Lynch et al., 1999). It is also possible that non‐captive baboons are still experiencing environments which are not entirely free from human influence, leading to anthropogenic changes in food availability. Eller et al. (2021) analyzed 875 NHP specimens in the NMNH collections and found that over 90% of the NHP specimens do not come from so‐called “wild” settings as often assumed. Instead, these animals lived in habitats within terrestrial biomes of varying degrees of anthropogenic influence, designated and mapped as “anthromes” by Ellis et al. (2010). The sample used by Eller et al. (2021) included 41 of the 51 baboon specimens that are present in the current study. Of the 41 specimens with anthrome information, most (95.1%) lived and died in seminatural, rangeland, and cropland localities in southern and eastern Africa (Eller et al., 2021; Supplementary Table 2). Within these environments, baboons likely consumed some amount of human food (Fehlmann et al., 2017), although it is unknown if and how these factors may have contributed to nutritional disorders in some cases. Future research could help to assess individual‐level factors contributing to MBD development in baboons or other NHPs in natural settings.

### 4.3 Skin color and dietary influences on MBD development

Diet and environment play an important role in mediating nutritional status. Recent evidence suggests that there are differences in initial cutaneous vitamin D metabolites between *Papio* species based on exposed facial skin pigmentation and sunlight conditions (Ziegler et al., 2018). A major limitation of the Ziegler et al. (2018) study was that UV exposure and diet were not controlled across the different baboon groups. Thus, our study examined whether differences in disease presentation between the six species were correlated with physiological differences associated with skin pigmentation. We found that this was not the case. While baboons display interspecies variability in skin pigmentation (Hill, 1967; Kamilar, 2006), this trait alone likely did not influence the presentation of MBD, which may instead reflect a combination of environmental factors.

Baboons may not be as reliant on sunlight exposure for vitamin D as other species, but rather are supplemented through dietary sources in their natural habitats. Most of their skin is covered in a thick pelage (regardless of color), which may hinder cutaneous vitamin D production. Further, they are known as hardy and diverse dietary generalists who can survive on a nonspecialized diet (Codron et al., 2006) and have been known to “raid” anthropogenic spaces for food items (Fehlmann et al., 2017). While some raids occur in more urbanized settings, such as Cape Town, South Africa (van Doorn & O'Riain, 2020), most are crop raids targeting small farming settlements across Africa (Hill, 2000; Kifle & Bekele, 2020; Maples et al., 1976; Warren, 2009). Because of their ability to exploit a variety of natural and anthropogenic food sources, baboons may be able to effectively supplement their nutritional needs through foraging behaviors.

Inadequate levels of vitamin D, calcium, and phosphorus may have all contributed to the prevalence of MBD. While calcium and phosphorus have known dietary sources for NHPs (fruit and leaves from uncultivated plants; Milton, 2003), sources of vitamin D are less understood. Vitamin D may derive from three natural dietary sources: mushrooms, meat, and insects. Mushrooms are known sources of vitamin D_2_ (Jasinghe & Perera, 2005), and *P. cynocephalus* at Mukumi National Park (Tanzania), for example, are known to utilize these food sources often (Norton et al., 1987). However, most mammals are unable to process vitamin D when it is in the form of vitamin D_2_ and so mushrooms may not contribute much to the vitamin D requirements for baboons (Horst et al., 1990). Meat, particularly fish, fatty livers, and egg yolks, is the best source of dietary vitamin D aside from natural production via UV radiation (Schmid & Walther, 2013).

Meat eating is known in most of the baboon species at sites across Africa (Goffe & Fischer, 2016; Morris & Goodall, 1977; Rhine et al., 1986; Schreier et al., 2019). It is unclear from behavioral observations if baboons prefer certain body parts or organs to others, since prey taxonomy is usually the only information reported for baboon meat consumption. However, Rhine et al. (1986) observed eggs being consumed by *P. cynocephalus* at Mukumi National Park, which may indicate a prominent dietary source of vitamin D. Finally, insects are sometimes included in baboon diets, as observed in locust and dragonfly consumption by *P. cynocephalus* and *P. hamadryas* troops (Rhine et al., 1986; Schreier et al., 2019). Locusts, along with a subset of other insect taxa, have an especially high vitamin D_3_ content for body size (Oonincx et al., 2010).

### 4.4 Limitations and future directions

The primary limitations of this study are the low availability of postcranial remains, which may have aided MBD identification in younger individuals, and incomplete documentation of the life history of the animals in the sample. Furthermore, there are many aspects of the captive experience that are critical to the story of the captive animal. Was it born in captivity or captured in the wild? At what age was it captured? How was it transported and for how long was it in transit? What was the captive environment like, and did it change throughout the life of the animal? To the best of our abilities, we utilized all available records to incorporate aspects of the animal's life into the design of our study. However, such information is scarce for individuals within the temporal span of our sample. Future work investigating modern populations will do well to incorporate such life history data.

Just like with the shortcomings of diet, living conditions, and veterinary care in historically captive environments (Gutierrez et al., 2021), non‐captive baboons may experience the same nutritional deficiencies that lead to the development of MBD. It is likely that skin pigmentation and sunlight exposure are not the only factors in MBD development; dietary imbalances may be just as influential. Gastrointestinal parasites may also play a role in MBD etiology by altering nutrient uptake, but there is sparse literature on the subject. This possibility warrants further investigation. Coping with extreme anthropogenic settings, like historically captive and urbanized environments, likely leads to more frequent or severe disease outcomes. As with current trends in human health research (Stearns, 2012), investigating how NHP biology responds to different environmental conditions is a valuable ecological perspective on disease for veterinary and conservation science.

## CONFLICT OF INTEREST

The authors declare no conflict of interest in the publication of this study.

## AUTHOR CONTRIBUTIONS

**Srishti Sadhir:** Conceptualization (equal); data curation (lead); formal analysis (lead); methodology (equal). **Andrea Eller:** Conceptualization (equal); data curation (supporting); formal analysis (supporting); methodology (equal). **Stephanie Canington:** Conceptualization (equal); data curation (supporting); formal analysis (supporting); methodology (equal). **Sabrina Sholts:** Conceptualization (equal); data curation (supporting); formal analysis (supporting); methodology (equal).

## Supporting information


**Supplementary Table 1.** NMNH baboon skull specimens (N = 160) with all demographic and pathological information.
**Supplementary Table 2.** Geographic distribution of non‐captive baboon sample from known modern‐day African nations and localities (n = 72). Regions follow those denoted by the African Union (AU). Names in parentheses and italics are historical, colonial names. Specimens were deemed “non‐captive” if individuals were born and lived in their native African range at the time of collection.
**Supplementary Table 3.** Accession year groupings by *Papio* species.
**Supplementary Table 4.** Diagnosis of pathological specimens (n = 64) using MBD criteria from Table 2.

## Data Availability

The data that support the findings of this study are available in the supporting information of this article and at OSF (https://doi.org/10.17605/OSF.IO/U4J3N).
